# Change in patellar height in medial and lateral unicompartmental knee arthroplasty: a clinical trial

**DOI:** 10.1007/s00402-023-05139-8

**Published:** 2023-12-18

**Authors:** Riccardo D’Ambrosi, Francesco Rubino, Chiara Ursino, Ilaria Mariani, Nicola Ursino, Matteo Formica, Julia Prinz, Filippo Migliorini

**Affiliations:** 1 IRCCS Ospedale Galeazzi Sant’Ambrogio, Milan, Italy; 2 Dipartimento di Scienze Biomediche per la Salute, Università degli Studi di Milano, Milan, Italy; 3 Orthopaedic Clinic, IRCCS Hospital Policlinico San Martino, Genoa, Italy; 4 Institute for Maternal and Child Health IRCCS Burlo Garofolo, Trieste, Italy; 5 DISC - Department of Surgical Sciences and Integrated Diagnostics, University of Genoa, Genoa, Italy; 6 Department of Ophthalmology, RWTH University Hospital, 52074 Aachen, Germany; 7 Department of Orthopaedic, Trauma, and Reconstructive Surgery, RWTH University Hospital, Pauwelsstraße 30, 52074 Aachen, Germany; 8 Department of Orthopaedics and Trauma Surgery, Academic Hospital of Bolzano (SABES-ASDAA), Teaching Hospital of the Paracelsus Medical University, 39100 Bolzano, Italy

**Keywords:** Unicompartmental knee arthroplasty, UKA, Patella height, Insall–Salvati, Caton–Deschamps

## Abstract

**Introduction:**

Evidence on patellar height changes following unicompartmental knee arthroplasty (UKA) is lacking. Therefore, this study compared the patella height in patients who underwent medial versus lateral UKA. Moreover, a subgroup analysis was conducted to investigate whether sex, age, and BMI of the patients exert an influence on the postoperative patellar height.

**Methods:**

Radiographs and hospital records of patients undergoing UKA were prospectively collected. Surgeries were performed by one author with long experience in UKA in a highly standardised fashion. The implants were fixed-bearing medial PPK (Zimmer Biomet, Warsaw, Indiana, USA) and fixed-bearing lateral ZUK (Lima Corporate, Udine, Italy). The patellar height was measured using the Insall–Salvati and Caton–Deschamps indices.

**Results:**

A total of 203 patients were included: 119 patients were included in the medial and 84 in the lateral UKA. The mean age of the patients was 68.9 ± 6.7 years, and the mean BMI was 28.1 ± 4.1 kg/m^2^. 54% (110 of 203 patients) were women. On admission, between-group comparability was found in age, BMI, sex, and length of the follow-up. No between-group and within-group difference was detected pre- and post-operatively in the Insall–Salvati and Caton–Deschamps indices in patients who have undergone medial versus lateral UKA. Concerning the subgroup analyses, no between-group and within-group difference was detected pre- and post-operatively in all comparisons according to sex, age, and BMI.

**Conclusion:**

No difference was found in patella height in patients who have undergone medial compared to lateral UKA. Furthermore, there was no evidence of an association between patient characteristics (sex, age, BMI) and patella height between medial and lateral UKA.

## Introduction

Osteoarthritis of the knee is a major health issue [1–3]. Unicompartmental knee arthroplasty (UKA) is a well-established treatment option in patients with medial or lateral unicompartmental OA since its introduction in 1975 [4–6]. UKA recreates joint space in the affected compartment [7–10]. Compared to total knee arthroplasty (TKA), the cruciate ligaments and extensor mechanism are spared in UKA, ensuring more physiologic kinematics [11–13]. UKA is associated with faster recovery, shorter hospitalisation, lower intraoperative blood loss, better functional outcomes and patient satisfaction, and greater postoperative range of motion than TKA [1, 14, 15]. Given the physiological alignment of the lower limb, approximately 90% of unilateral OA and UKA are at the medial compartment [16–20]. Contraindications of UKA include high BMI, deformities, and restricted joint motion [21].

Patella height changes following TKA might result in anterior knee pain, midflexion instability, and reduced postoperative range of motion [22–25]. Lower BMI, women, and older age have been associated with a greater risk of patella height following TKA [26]. Other acquired risk factors for patellar height changes following TKA have been described, including removal of Hoffa’s fat pad, scar tissue formation, intraoperative over-resection of the distal femur, and the type of surgical approach [27–32]. However, evidence on patellar height changes following UKA is lacking [33].

The present study compared the patella height in patients who underwent medial versus lateral UKA for UKA. Moreover, a subgroup analysis was conducted to investigate whether sex, age, and BMI of the patients exert an influence on the postoperative patellar height.

## Materials and methods

This study was conducted following the Strengthening the Reporting of Observational Studies in Epidemiology (STROBE) statement [34]. All procedures involving human participants in this study were performed in accordance with the ethical standards of the institutional and/or national research committee as well as the 1964 Helsinki Declaration and its later amendments or comparable ethical standards. Informed consent was obtained from all the participants. The present study was approved by the Ethics Committee of the San Raffaele University Hospital of Milan, Italy (CE 236/2017). Data from patients who have undergone elective primary UKA at our institution between 2018 and 2021 were prospectively collected.

The indication for surgery was isolated unicompartmental symptomatic OA grades III to IV according to the Kellgren–Lawrence rating score [35]. In all the cases, the anterior cruciate, medial collateral, and lateral collateral ligaments were functionally intact, as confirmed by magnetic resonance imaging (MRI) and clinical examination. Patients who had previous surgery of the affected knee (except arthroscopy for meniscectomy), varus/valgus deformities, flexion deficit greater than 15°, and insufficient anterior or posterior cruciate ligament were considered as exclusion criteria.

### Procedures

Surgery was performed by one author with long experience in UKA in a highly standardised fashion. The implants were fixed-bearing medial PPK (Zimmer Biomet, Warsaw, Indiana, USA) and fixed-bearing lateral ZUK (Lima Corporate, Udine, Italy). All patients were placed in a supine position on a standard operating table under spinal anaesthesia. A standard medial or lateral parapatellar approach was used. Inspection of the patellofemoral and medial compartments was performed. All components were cemented using Refobacin Bone Cement R (Zimmer Biomet, Warsaw, Indiana, USA). An intraarticular drain was placed and removed on the first postoperative day. Enoxaparin sodium 4000 units subcutaneously daily for 45 days was used as thromboembolic prophylaxis.

The postoperative protocol was conducted following a previous report [36]. Briefly, both the patient groups followed the same rehabilitation protocol involving passive mobilisation from day one after the surgery. From day two, they started an active progressive mobilisation of the joint and assisted walking with two crutches. According to each patient’s capability, a gradual increase in the load during walking was recommended, continuing with isometric muscle toning exercises until the total abandonment of walking aids.

### Assessment

**Fig. 1:**
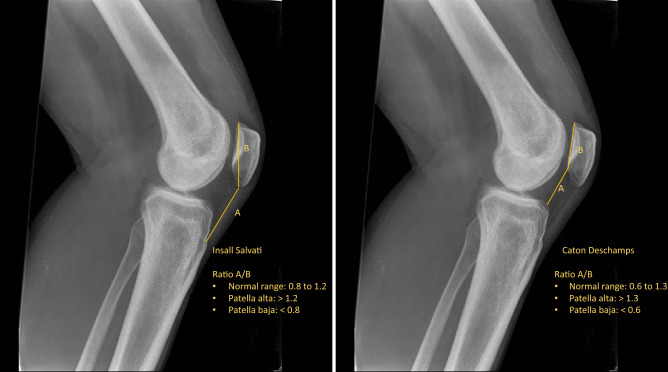
Methods to evaluate the IS and CD on plain radiographs

Two independent observers (experienced orthopaedic surgeons) assessed the preoperative and minimum 24-month postoperative radiographs. The radiographic measurements were performed twice with an interval of a minimum of 4–6 weeks. The standardised postoperative radiographs were aligned under fluoroscopic guidance to obtain views parallel to the tibial component in the anteroposterior view and parallel to the femoral component in the lateral view with the knee flexed at 30° without weight-bearing [37, 38]. Data concerning patients' age, sex, BMI, and location of the UKA (medial or lateral) were prospectively collected. The patellar height was measured using the Insall–Salvati (IS) [39] and Caton–Deschamps (CD) [40] indices. Both indices are validated methods to assess patellar height in the lateral projection of the knee at 30° of flexion (Fig. 1) [41–43].

### Power analysis

An estimated sample of 71 subjects for each group was required to compare patellar height between medial and lateral UKA position with a two-sided Wilcoxon–Mann–Whitney test, given an index mean difference of 5, a standard deviation of 8 for both groups, a 5% alpha, and a 95% power. This sample had also a 99% power to detect a difference between pre- and postoperative values with a one-sided Wilcoxon signed-rank test, assuming a mean difference of 5, a standard deviation of 8 for both groups, and a 2.5% alpha. Additional subjects were recruited to ensure statistical significance in case of adverse events.

### Statistical analysis

Statistical analyses were conducted in R version 4.1.1 (R Development Core Team, Austria) and SAS/STAT version 9.3 (SAS Institute Inc., Cary, NC, USA). Summary statistics are presented as mean and standard deviation for continuous data or absolute frequency and percentage for dichotomic data. A chi-square test for categorical variables and, for continuous variables, a *t*-test (for parametric statistics), or a Wilcoxon–Mann–Whitney test (for non-parametric statistics) was performed. To compare CD and IS, a *t*-test or a Wilcoxon signed ranks test was conducted based on score distribution. Subgroup analysis was performed to evaluate whether gender, age, and BMI are associated with differences in patellar height. Both in the intergroup and the subgroup analysis, age and BMI groups were defined dichotomizing the variable at its average rounded value, while pre-operatory values of CD and IS above and below 1 were used to identify patients with low and high patellar position.

## Results

### Patient enrolment

**Fig. 2:**
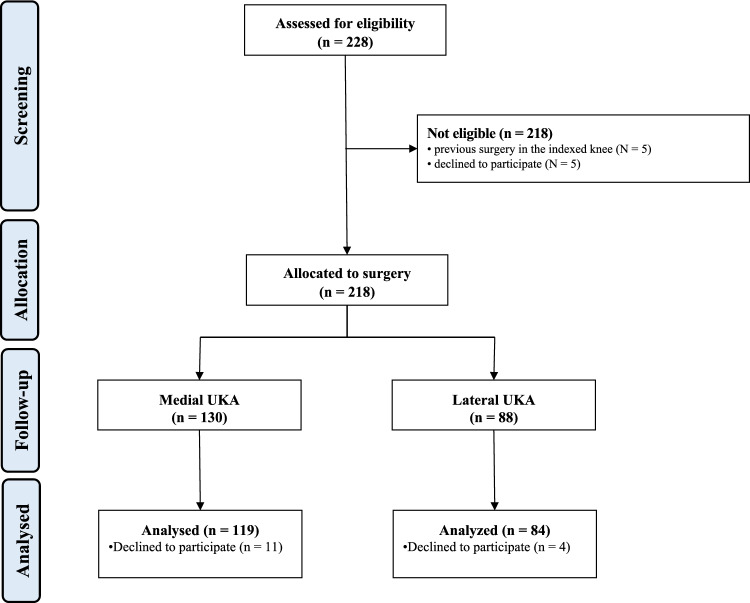
STROBE flow chart of patient enrolment

228 patients were recruited. Of them, 5 patients (*N* = 3 anterior cruciate ligament reconstruction, *N* = 2 tibial osteotomy) were excluded as they had undergone previous surgery on the knee. A further five patients declined to participate. 223 patients underwent surgery. 15 patients were lost at follow-up. This left 203 patients for inclusion: 119 patients were included in the medial UKA and 84 in the lateral cohort (Fig. 2).

### Patient demographic

The mean age of the patients was 68.9 ± 6.7 years and the mean BMI was 28.1 ± 4.1 kg/m^2^. 54% (110 of 203 patients) were women. On admission, between-group comparability was found in age, BMI, sex, and length of the follow-up (Table 1).

**Table 1:** Demographic data

Endpoint	Medial (*N* = 119)	Lateral (*N* = 84)	*P*
Age	69.69 ± 7.25	67.89 ± 5.77	0.07
BMI	27.91 ± 4.25	27.85 ± 3.96	0.9
Women	65 (54.6%)	45 (53.6%)	0.9
Follow-up (months)	26.9 ± 1.8	26.7 ± 1.7	0.4

### Results syntheses

No between- and within-group difference was detected pre- and post-operatively in the Insall–Salvati and Caton–Deschamps indices of patients who underwent medial and lateral UKA (Table 2).

**Table 2:** Pre- and postoperative radiographic and clinical scores

	Medial (*N* = 119)	Lateral (*N* = 84)	*P*
CD pre-op	0.97 ± 0.15	0.98 ± 0.14	0.691
CD post-op	0.95 ± 0.14	0.99 ± 0.15	0.058
IS pre-op	1.05 ± 0.15	1.08 ± 0.14	0.129
IS post-op	1.04 ± 0.15	1.06 ± 0.13	0.547

IS, Insall–Salvati; CD, Caton–Deschamps

### Subgroup analysis

No between- and within-group difference was detected pre- and post-operatively in all comparisons according to sex, age, and BMI (Table 3).

**Table 3:** Subgroup analysis between medial and lateral UKA divided for gender, age, and BMI

Women	Medial	Lateral	*P*
	*N* = 45	*N* = 65	
CD pre-op	0.97 ± 0.15	0.99 ± 0.14	0.6
CD post-op	1.01 ± 0.17	0.97 ± 0.14	0.3
IS pre-op	1.10 ± 0.14	1.06 ± 0.15	0.08
IS post-op	1.07 ± 0.13	1.06 ± 0.13	0.9

Men	*N* = 39	*N* = 54	
CD pre-op	0.98 ± 0.13	0.95 ± 0.16	0.4
CD post-op	0.97 ± 0.13	0.93 ± 0.14	0.1
IS pre-op	1.06 ± 0.15	1.03 ± 0.15	0.7
IS post-op	1.05 ± 0.14	1.02 ± 0.16	0.3

Age < 70 years	*N* = 40	*N* = 51	
CD pre-op	0.99 ± 0.15	0.97 ± 0.16	0.5
CD post-op	0.99 ± 0.15	0.97 ± 0.16	0.5
IS pre-op	1.08 ± 0.13	1.04 ± 0.17	0.7
IS post-op	1.06 ± 0.13	1.03 ± 0.16	0.7

Age ≥ 70 years	*N* = 44	*N* = 68	
CD pre-op	0.96 ± 0.13	0.97 ± 0.14	0.5
CD post-op	0.99 ± 0.16	0.94 ± 0.13	0.1
IS pre-op	1.08 ± 0.16	1.05 ± 0.13	0.1
IS post-op	1.06 ± 0.14	1.05 ± 0.14	0.8

BMI < 28	*N* = 44	*N* = 68	
CD pre-op	0.96 ± 0.11	0.96 ± 0.16	0.7
CD post-op	0.96 ± 0.15	0.95 ± 0.13	0.8
IS pre-op	1.08 ± 0.17	1.04 ± 0.15	0.6
IS post-op	1.05 ± 0.15	1.05 ± 0.14	0.9

BMI ≥ 28	*N* = 40	*N* = 51	
CD pre-op	1.00 ± 0.16	0.98 ± 0.13	0.5
CD post-op	1.03 ± 0.15	0.96 ± 0.16	0.06
IS pre-op	1.09 ± 0.11	1.05 ± 0.15	0.09
IS post-op	1.07 ± 0.11	1.04 ± 0.16	0.3

IS, Insall–Salvati; CD, Caton–Deschamps

## Discussion

This study prospectively compared the patella height in patients who underwent medial versus lateral UKA. The prospective design might contribute to reduce the risk of selection bias, impacting the reliability of the results. According to the main findings of the present study, no difference was found in patella height between patients who underwent medial compared to lateral UKA. Furthermore, there was no evidence of an association between patient characteristics (sex, age, BMI) and differences in patella height between the two implants. These results agree with previous investigations which found inconsistent results [8, 16, 44]. However, no superiority of medial versus lateral UKA survival was found in a previous systematic review with a follow-up of 15 years and a previous meta-analysis reporting the 10-year outcomes [8, 45]. Weale et al. [46] found no significant change in patellar height up to 5 years following UKA. These results might be explained by the resurfacing nature of the procedure and the limited bone cuts, and changes in patellar height after UKA are limited. Moreover, the minimally invasive incision and the avoidance of patellar eversion cause less trauma to Hoffa’s fat pad and also prevent changes in patellar height [47].

UKA is a bone- and ligament-sparing option in patients with unicompartmental OA [7, 45]. Approximately 90% of all UKA procedures are performed at the medial and 10% at the lateral compartment [16, 17], which is attributable to a higher incidence of diseases, such as OA or osteonecrosis, at the medial compartment [8]. Compared to medial UKA, lateral UKA is considered more challenging given the anatomic and kinematic differences between the two compartments [17, 48]: in contrast to the medial femoral condyle, the lateral condyle shows backwards rolling and sliding during flexion [48, 49]. Furthermore, implant design factors and the lower surgical volume in lateral compared to medial UKA might contribute to the higher technical complexity in lateral UKA [17, 50]. In addition, the patella tracks more laterally during high flexion, possibly causing patellar impingement [17]. The patellar height affects the knee biomechanics and patellofemoral function [51–53]. Recently, the importance of patellar height following knee surgery, especially TKA, has been discussed [22, 54]. Both IS and CD are validated and well-known methods to evaluate patellar height [55–57]. Different methods to measure patellar height have been developed over time [33, 58, 59]. Verhulst et al. [38] found that the IS ratio but not the CD ratio was associated with a high intra- and inter-observer reliability. In contrast, good intra- and inter-observer reliability for both the IS and the CD ratio was found in other studies [60, 61].

Postoperative patella baja refers to a decreased patellar height and might occur following the shortening of the patellar tendon due to scar formation and contracture, and pseudo-patella baja happens in joint line elevation [62]. Joint line elevation possibly results in a reduction of the extensor mechanism power and a reduced knee flexion due to a tightening of the collateral ligaments and reduced femoral rollback [63]. Moreover, joint line elevation was associated with anterior knee pain, patellofemoral problems, reduced knee flexion capability, and midflexion joint instability in previous studies [63–65]. Patella baja following TKA has been investigated in numerous studies, while its occurrence in patients undergoing UKA is still unclear to date [47].

## Conclusion

No difference was found in patella height between patients who underwent medial compared to lateral UKA. No evidence of an association between patient characteristics (sex, age, BMI) and patella height between the two implants was found.

## Data Availability

All data and materials are available on reasonable request to Dr. Riccardo D’Ambrosi (riccardo.dambrosi@hotmail.it).

## Abbreviations

BMI: Body mass index
IS: Insall–Salvati
CD: Caton–Deschamps
UKA: Unicompartmental knee arthroplasty
OA: Osteoarthritis
STROBE: Strengthening the Reporting of Observational Studies in Epidemiology
